# Temperature-Responsive Injectable Composite Hydrogels Based on Poly(*N*-Isopropylacrylamide), Chitosan, and Hemp-Derived Cellulose Nanocrystals

**DOI:** 10.3390/polym16212984

**Published:** 2024-10-24

**Authors:** Praewa Promdontree, Artjima Ounkaew, Yuan Yao, Hongbo Zeng, Ravin Narain, Sarute Ummartyotin

**Affiliations:** 1 Department of Materials and Textile Technology, Faculty of Science and Technology, Thammasat University, Pathumthani 12121, Thailand; pwpraewapwp@gmail.com; 2 Department of Chemical and Materials Engineering, University of Alberta, Edmonton, AB T6G 2G6, Canada; ounkaew@ualberta.ca (A.O.); yyao24@ualberta.ca (Y.Y.); hongbo.zeng@ualberta.ca (H.Z.); 3 Center of Excellence on Petrochemical and Materials Technology, Chulalongkorn University, Bangkok 10330, Thailand

**Keywords:** hemp cellulose, cellulose nanocrystals, Poly(*N*-Isopropylacrylamide), chitosan, photopolymerization, thermo-responsive injectable hydrogel, cytotoxicity

## Abstract

Injectable and temperature-responsive Poly(*N*-Isopropylacrylamide) (PNIPAAm)/Chitosan composite hydrogels reinforced with cellulose nanocrystals (CNCs) were successfully fabricated via photopolymerization. 0.1–3% (*w*/*v)* of cellulose nanocrystals were incorporated into the PNIPAAm/chitosan matrix to form thermo-responsive injectable composite hydrogels. FT-IR spectra confirmed the successful formation of these hydrogels, highlighting the characteristic peaks PNIPAAm, chitosan and CNCs. The inclusion of CNCs led to a reduced pore size as compared to the control hydrogels. The mechanical properties of the hydrogel were characterized under various temperature conditions. Rheology tests showed that storage modulus (G′) increased significantly above 30 °C, indicating gel-like behavior. Thermogravimetric analysis showed thermal stability up to 300 °C. The volume phase transition temperatures (VPTT) of the hydrogels were found to be in the range of 34–38 °C, close to physiological body temperature. The equilibrium swelling ratio (ESR) of the CNC-containing hydrogels was higher than that of the control. In vitro studies with Human Dermal Fibroblast adult (HDFa) cells showed the hydrogels to be non-toxic, suggesting their potential for biomedical applications.

## 1. Introduction

In recent years, various types of low cost medical and pharmaceutical technologies have been developed to cope with the growing worldwide population. The goal is to improve access of advanced technologies such as chemical sensors for health monitoring in remote areas, wound dressings for various kinds of injuries as well as targeted delivery systems. Many of those technologies relied on hydrogels, a three-dimensional network composed of hydrophilic polymers that can retain a large amount of water. Hydrogels can either swell or contract under various chemical and physical conditions. The fabrication of hydrogels can be achieved by chemical and physical crosslinking throughout the polymer network [1,2]. Furthermore, it can be formulated into many physical forms such as slabs, films, coating, microparticles, and nanoparticles. Therefore, the properties of hydrogel can be tuned for numerous categories of biomedical research including tissue engineering, regenerative medicine, cell encapsulation, separation of biomolecules or cells, and barrier materials to prevent any infections [3,4]. Furthermore, ‘smart’ hydrogels can respond to various types of external stimuli such as temperature, electric and magnetic field, light, pressure as well as sound [5].

Most of the hydrogels were developed from petrochemical-based materials. Some examples of the most popular hydrogels are polyvinyl alcohol (PVA) [6,7], polyacrylamide (PAM) [8,9] as well as polyacrylic acid (PAA) [10,11]. Although they offer significant benefits such as dimensional stability as well as size/shape control, they are not easily degraded and there are some concerns for usage due to potential toxicity. To overcome these limitations, hydrogel was developed from bio-based materials [12,13]. Recently, one of the most attractive bio-based materials is related to chitosan and its derivatives. Chitosan is a naturally occurring abundant biopolymer on earth. It is structurally defined as a linear polysaccharide composed of randomly distributed β-(1→4)-linked D-glucosamine and N-acetyl-D-glucosamine [14,15]. It can be chemically extracted from shells of shrimp and other crustaceans. Chitosan has mucosal adhesion property, antibacterial activity, and biocompatibility. Recently, Lee et al. [16] developed carboxymethyl chitosan for a blood-contacting device which shows antimicrobial and antithrombotic performance. The functionalization enhanced the electrostatic repulsion for antifouling properties. Narciso et al. [17] fabricated a 3D-printed biosurfactant-chitosan coating for silicone which has antibacterial and antifouling properties. However, pristine chitosan has limitation for usage due to its low mechanical properties.

To overcome this issue, the development of chitosan and Poly(*N*-Isopropylacrylamide) based hydrogel composite was prepared. The composite hydrogel showed better mechanical properties as well as improved biocompatibility. Recently, in 2022, Romero et al. [18] developed Poly(*N*-Isopropylacrylamide) and chitosan as a template for silver nanoparticle formation. The temperature responsive behavior of Poly(*N*-Isopropylacrylamide) helped with the synthesis of silver nanoparticles with controllable size/shape. Moradi et al. [19] fabricated Fe_3_O_4_ containing Poly(*N*-Isopropylacrylamide)-grafted onto chitosan. The nanocarrier has both pH and temperature-responsive properties suitable for delivery of anticancer drugs. Li et al. [20] studied multi-responsive of Poly(*N*-Isopropylacrylamide)/poly(*N*-*tert*-butylacrylamide)/chitosan composite film which inhibitor the growth of Gram-positive and Gram-negative bacteria.

Injectable hydrogel offered various benefits including minimal invasiveness and achieving localized release into targeted organs [21,22,23]. Wiwatsamphan et al. [24] developed Poly(*N*-Isopropylacrylamide) and chitosan composite hydrogel via thiol-ene “Click” reaction which revealed to be responsive to both pH and temperature. Hoang et al. [25] also developed dual pH-/thermo-responsive chitosan-Poly(*N*-Isopropylacrylamide) based hydrogel via “Click chemistry”. A high encapsulation of drug can be achieved and released of the cargo was achieved under weak basic conditions. Shi et al. [26] developed thermo-responsive injectable hydrogel from carboxymethyl chitosan and sodium alginate particles showing non-toxicity and excellent hemocompatibility for bone regeneration.

Furthermore, in order to enhance the property of injectable chitosan-based hydrogel composite, the integration of purified cellulose into hydrogel was considered as an alternative route. Cellulose is one of the most abundant naturally occurring bio-based polymers on earth. It offered various advantages such as high stiffness, high chemical resistance as well as high dimensional stability [27]. Liu et al. [28] fabricated injectable hydrogel from silk fibroin peptide grafted onto hydroxypropyl chitosan and oxidized microcrystalline cellulose for wound healing. It offered high antioxidant activity and good biocompatibility. Ghorbani et al. [29] developed cellulose and chitosan-based hydrogel composite. The incorporation of cellulose into the chitosan matrix offered cell proliferation as well as high resistance to degradation. One of the most important resources for cellulose production was focused on hemp-based cellulosic materials [30]. Mahur et al. [31] prepared cellulose-based material with an additional feature of morphology from hemp. It can be prepared into web-like, rod-like as well as needle-like shapes based on various concentrations of sulfuric acid.

In this research work, we prepare injectable composite hydrogel from chitosan and Poly(*N*-Isopropylacrylamide). Purified cellulose nanocrystals from hemp were integrated into the hydrogel matrix. Fourier transform infrared and thermogravimetric analysis were employed to evaluate the structural and thermal properties, respectively. Volume phase transition temperature was investigated to identify the lower critical solution temperature for sol-gel phase transition. Microstructural properties were determined by scanning electron microscope. The hydrogels were subsequently evaluated for injectability, mechanical properties, its swelling behavior and cytotoxic test.

## 2. Experimental

### 2.1. Materials and Chemical Reagents

The dried hemp fiber (*Cannabis sativa*) was received from a local farm in Thailand. It was ground by nano-grinder and stored in a desiccator. The monomer of *N*-isopropylacrylamide (NIPAAm, M_w_ = 113.16 g/mol) was purchased from Tokyo Chemical Industry Co., Ltd. (Tokyo, Japan). Chitosan (M_w_ = 50,000–190,000 g/mol) was purchased from Biosynth International, Inc. (Louisville, Kentucky, USA). Sodium hydroxide (NaOH), glacial acetic acid (CH_3_COOH) and sodium chlorite (NaClO_2_) were purchased from Merck, Co, Ltd. (Bangkok, Thailand), M.S. Chemical Co, Ltd. (Bangkok, Thailand) and DC Fine Chemicals, Ltd. (Enfield, UK), respectively. Sulfuric acid (H_2_SO_4_) was purchased from QRëC™ (Quality Reagent Chemical), Co, Ltd. (Chonburi, Thailand). Irgacure-2959 (I-2959) was obtained from Ciba Inc. (Basel, Switzerland). Phosphate buffered saline (PBS; pH 7.4) was purchased from Cytiva Co, Ltd. (Marlborough, MA, USA). The 23G needles and 10 mL syringes were purchased from Becton, Dickinson and Company, Co. Ltd. (Franklin Lakes, New Jersey, USA). All chemical reagents were analytical grade and used as received without further purification.

### 2.2. Methodology

#### 2.2.1. Preparation of Cellulose Nanocrystals

The cellulose microfiber was extracted from the hemp fiber with alkali treatment. The experiment was based on the protocol of Zhao et al. [32] with modifications. Briefly, the raw hemp fiber was first dried at 65 °C for 24 h and ground using a nano-grinder (High Energy Ball Mills, Emax, Germany) to decrease its size. Then, it was treated with 18% (*w*/*v*) of NaOH at 80 °C for 2 h under mechanical stirring. After that, it was bleached 2 times with acetate buffer. The buffer was prepared by adding 2.7 g of NaOH and 7.5 mL of glacial acetic acid into 100 mL of distilled water. Then, 2% (*w*/*v*) of NaClO_2_ was poured into the mixture, and the solution was stirred at 80 °C for 2 h. After that, it was washed with distilled water until a neutral pH was achieved. The samples were stored in the suspension form. Additional information was presented in our previous literature [33].

Cellulose nanocrystals (CNCs) were successfully extracted with sulfuric acid hydrolysis based on the guidance of Leong et al.’s protocol [34]. Briefly, the CMF was treated with 0.9 M of sulfuric acid solution at 50 °C for 15 min under mechanical stirring. Then, it was quenched with cold distilled water for 15 min using a probe-type ultrasonic homogenizer. After that, DI water was used to adjust the pH of the suspension to neutral pH before additional usage. The centrifugation was set to a rotation speed of 900 rpm for 5 min. Additional information was presented in the previous literature [33].

#### 2.2.2. Preparation of Thermo-Responsive Composite Hydrogels with Poly(*N*-Isopropylacrylamide), Chitosan and Cellulose Nanocrystals from Hemp 

**Figure 1:**
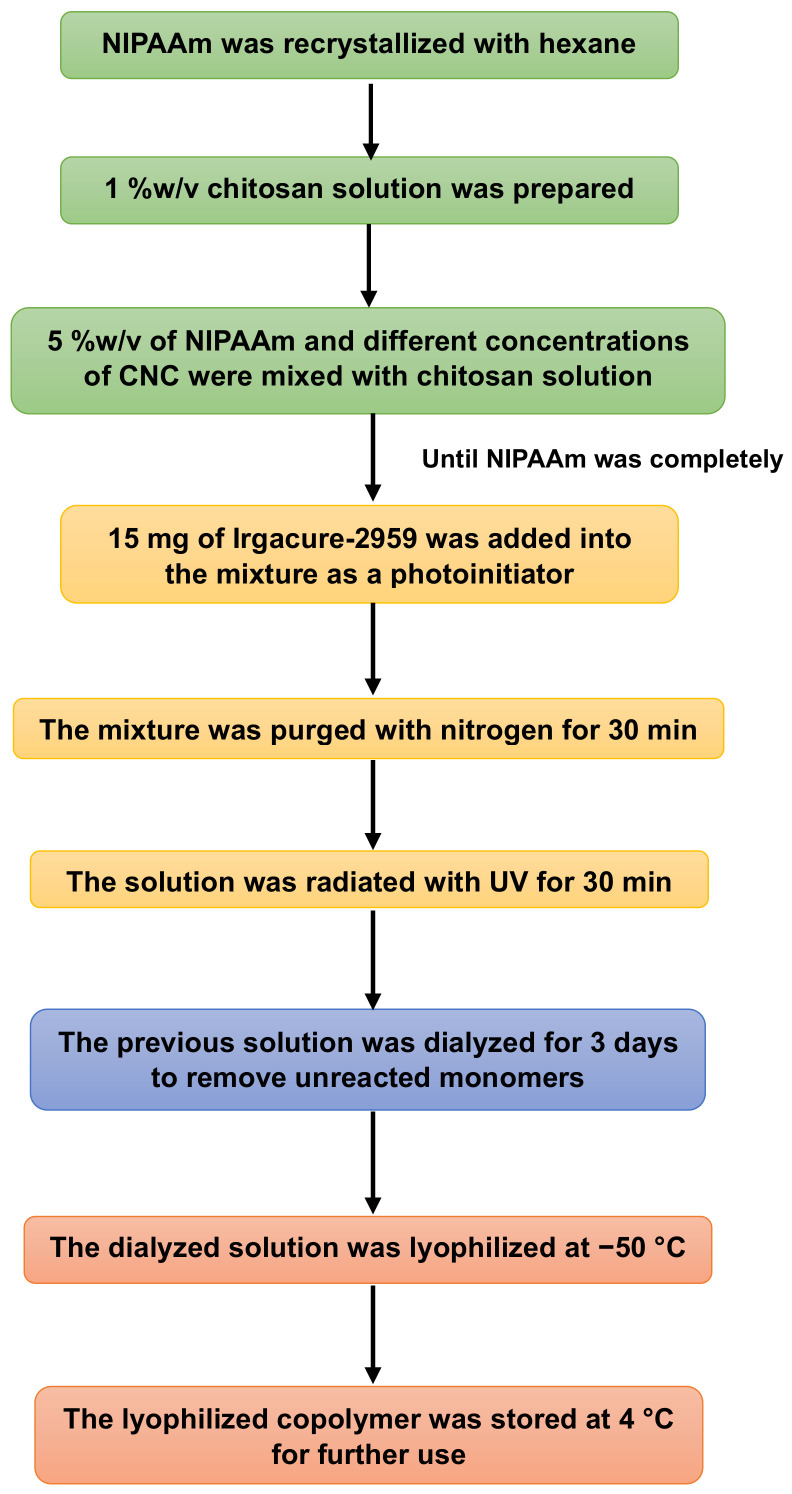
Schematic diagram of the preparation of injectable composite hydrogel based thermo-responsive Poly(*N*-Isopropylacrylamide), chitosan and cellulose nanocrystals from Hemp.

The NIPAAm monomer required recrystallization before use. First, hexane at 60 °C was carefully added into NIPAAm (ratio: 100 mL/10 g). The solution was placed in a fume hood for 24 h. The precipitated NIPAAm was then filtrated by vacuum filtration, dried and stored at −4 °C. Thermo-responsive PNIPAAm/Cs/CNCs injectable hydrogels were prepared via photopolymerization. Initially, 100 mg of chitosan was dissolved in 10 mL of 0.1 M aqueous acetic acid at room temperature overnight under magnetic stirring. Then, 5%*w*/*v* of NIPAAm and different concentrations of CNCs (10, 50, 100, 150, 300 mg) were mixed with chitosan solution. Subsequently, 15 mg of Irgacure-2959 was added to the mixture. The mixture was purged with nitrogen for 30 min before photopolymerization. After that, the mixture was irradiated with UV (LZC Photoreactor with UV lamp, Luzchem Research Inc., Ottawa, ON, Canada) for 30 min. The solution was dialyzed by DI water for 3 days to remove unreacted monomers and subsequently lyophilized at −50 °C by freeze-dryer (FreeZone Freeze Dryers, Labconco, Kansas City, MO, USA) for 48 h. The lyophilized samples were kept at 4 °C for further use. Sample without CNCs was prepared by a similar approach. Additional information is presented in Figure 1.

#### 2.2.3. Hydrogel Injection

The samples were dissolved in DI water to obtain 5% (*w*/*v*) solutions. Subsequently, the thermo-responsive hydrogels were injected through 23G needles on glass slides at 37 °C. The temperature-responsiveness of the composite hydrogels while injecting and after injection were photographed by a camera.

#### 2.2.4. Equilibrium-Swelling Ratio (ESR)

The freeze-dried composite hydrogels were used for the equilibrium-swelling ratio. The samples were prepared in equal sizes and weighed (W_d_) before being immersed in phosphate-buffered saline (PBS; pH 7.4) solution at temperatures (25, 37, and 45 °C). The immersed samples were allowed to swell for 24 h. The samples were removed from PBS and were carefully wiped with filter paper to remove excess surface solution. The equilibrium swollen samples were weighed (W_e_). The data was reported as statistical average and standard deviation. The equilibrium-swelling ratio (ESR, gg^−1^) in PBS was determined as follows equation

(1) $$ESR=\left(\frac{W_{e}-W_{d}}{W_{d}}\right)$$

where W_e_ is the weight of equilibrium swollen hydrogel at certain temperatures and W_d_ is the initial weight of the freeze-dried hydrogel.

#### 2.2.5. Cytotoxic Test

Freeze dried composite hydrogels were investigated for their toxicity via an extract test using adult human dermal fibroblasts (HDFa), as model cells. The freeze-dried samples were prepared in wells of a 96-well plate (Costar, Corning, Corning, NY, USA) in equal weight (1–2 mg). The thermo-responsive injectable hydrogels in the 96-well plate were sterilized with UV exposure for 45 min before culturing. The HDFa cells were placed into wells of a 96-well plate at a density of 1 × 10^4^ cells/well and cultured in complete medium. Then, the HDFa cells were incubated in a CO_2_ incubator (37 °C, 5% CO_2_) for 24 h. After that, the sterilized hydrogel samples were added to the previous HDFa cells culture. The cells were proliferated under a 37 °C humidified atmosphere containing 5% CO_2_ for 24 h. The blank without composite hydrogel was set as a control. After that, the samples were taken out from each well and carefully washed with PBS three times. The cytotoxicity was determined by the 3-(4,5-dimethylthiazol-2-yl)-2,5-diphenyltetrazolium bromide (MTT) assay. After 2–4 h of incubation in MTT solution (0.5 mg/mL PBS), the cells were stained. The excess MTT solution was subsequently removed, and dimethyl sulfoxide (DMSO) was added 100 µL to each well. Each well became purple due to the DMSO dissolving purple formazan crystals in viable cells. The cytotoxicity test was quantified using a microplate reader (Varioskan LUX multimode microplate reader, Thermo Fisher Scientific Inc. (Waltham, MA, USA) at an absorbance of 570 nm.

(2) $$\mathrm{Cell}\;\mathrm{viability}\;(\%)=\left(\frac{{\mathrm{OD}}_{570\;\mathrm{sample}}}{{\mathrm{OD}}_{570\;\mathrm{blank}(\mathrm{control})\;}}\right)\times \;100$$

### 2.3. Instruments

#### 2.3.1. Fourier Transform Infrared Spectroscopy

The samples were analyzed by using FT-IR (SHIMADZU Corporation, Kyoto, Japan) at room temperature. The FTIR spectra were recorded in the spectral range 4000–700 cm^−1^ with a resolution of 2 cm^−1^ and for 64 scans.

#### 2.3.2. Rheology Test

Hydrogel samples at 5% (*w*/*v)* were prepared for rheological analysis. The rheological properties were determined using a rheometer type AR-G2 (TA Instruments, New Castle, DE, USA). Parallel plate geometry (20 mm diameter) and with a gap of 1000 µm. After that, the solubilized composite hydrogel was loaded on the rheometer base and examined under the oscillation mode. Using the temperature sweep method, the G′ and G′′ were measured at a frequency of 1 Hz over a temperature range of 20 to 50 °C at a rate of 3 °C/min.

#### 2.3.3. Scanning Electron Microscopy

The samples were immersed into liquid nitrogen and fractured to investigate the cross-section. Then, the samples were coated with gold by a sputtering device (Hummer 6.2 Sputter Coater, Anatech Ltd., Michigan, USA). The scanning electron microscopy (EVO 10, ZEISS, Oberkochen, Germany) was photographed the microstructure in 400× magnification and 20 kV of accelerating voltage.

#### 2.3.4. Thermogravimetric Analysis

The samples were characterized by TGA (SDT Q600 TGA/DSC system, TA Instruments, New Castle, DE, USA). 5 mg of samples were placed in an aluminum pan. It was heated from 20 to 800 °C under nitrogen purge at a heating rate of 10 °C/min.

#### 2.3.5. Volume Phase Transition Temperature

The volume phase transition temperature (VPTT) was investigated by UV-vis spectrophotometer (V-630, Jasco, Easton, MD, USA) at 450 nm to determine the transmittance of each sample. Each hydrogel solution was heated in a disposable cuvette and the temperature was recorded. The solution at temperatures ranging between 20–44 °C, which was below and above the LCST, was immediately measured with a UV-vis spectrophotometer at 450 nm. The transmittance was calculated to absorbance (%) from the following equation:

(3) $$\mathrm{Absorbance}\;(\%)=\left(\frac{T_{t}-T_{0}}{T_{0\;}}\right)\times \;100$$

where T_0_ is the transmittance of the initial temperature and T_t_ is the transmittance of each point temperature of the thermo-responsive injectable hydrogel solutions.

## 3. Results and Discussion

### 3.1. Development of Cellulose Derived from Hemp and Polyvinyl Alcohol-Based Hydrogel Composite

All samples were prepared via photopolymerization from PNIPAAm and chitosan, also integrating CNCs as a reinforcement phase. The composites exhibited the characteristics of injectability and temperature stimuli to human body temperature which were optimized as wound dressings. Furthermore, the preparation of 5% (*w*/*v)* thermo-responsive injectable-based composite hydrogel solution was investigated to be the most effective for usage. This is similar to previous articles [35,36]. Irgacure-2959 was used as a photoinitiator to induce polymerization under UV irradiation [35,37].

**Figure 2:**
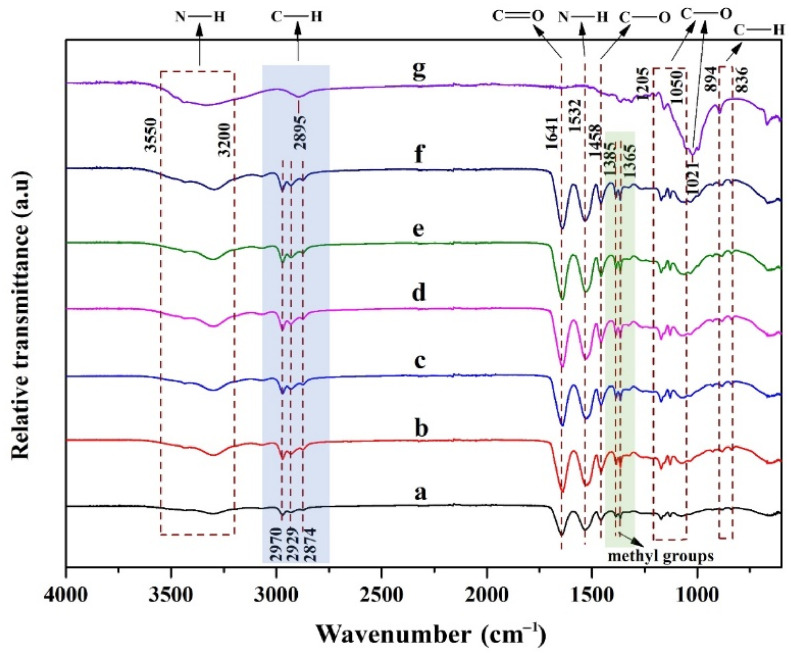
FT-IR spectra of thermo-responsive injectable hydrogels: (a) PNIPAAm/Cs/CNC0, (b) PNIPAAm/Cs/CNC10, (c) PNIPAAm/Cs/CNC50, (d) PNIPAAm/Cs/CNC100, (e) PNIPAAm/Cs/CNC150, (f) PNIPAAm/Cs/CNC300 and (g) Cellulose nanocrystals.

Figure 2 describes the FT-IR spectra of thermo-responsive injectable-based composite hydrogel. The broad peaks between 3550–3200 ${\mathrm{cm}}^{-1}$ were assigned as N–H stretching vibrations. The characteristic peaks of PNIPAAm were observed at 1641 ${\mathrm{cm}}^{-1}$ and 1532 ${\mathrm{cm}}^{-1}$ which are considered as C=O stretching vibration (amide I) and N–H bending vibration (amide II), respectively [35,36,37,38]. The absorption bands of PNIPAAm at the wavenumbers of 1385 ${\mathrm{cm}}^{-1}$, 1365 ${\mathrm{cm}}^{-1}$, and 2895 ${\mathrm{cm}}^{-1}$ were attributed to the stretching vibration of methyl groups CH(CH_3_)_2_ [35,37,38]. The characteristic peak at 1641 ${\mathrm{cm}}^{-1}$ was assigned to C=O stretching vibration of amide I in the chitosan [39,40]. Furthermore, this peak can be masked by the high content of PNIPAAm [35]. Several peaks in the ranges of 1205–1050 ${\mathrm{cm}}^{-1}$ were represented as the C–O stretch absorption of primary, secondary, and tertiary alcohols which were related to CNCs [37,41]. In this band, PVA and CNCs showed similarly characteristic absorption bands due to similar chemical groups, particularly hydroxyl (–OH) and C–O bonds [42]. As the comparison to FT-IR spectra of CNCs, the absorption bands between 2970–2874 ${\mathrm{cm}}^{-1}$ can be confirmed that the CNCs were successfully embedded into the matrix. Furthermore, the peak between 3550–3200 ${\mathrm{cm}}^{-1}$ was attributed to O–H stretching vibrations of the CNCs.

**Figure 3:**
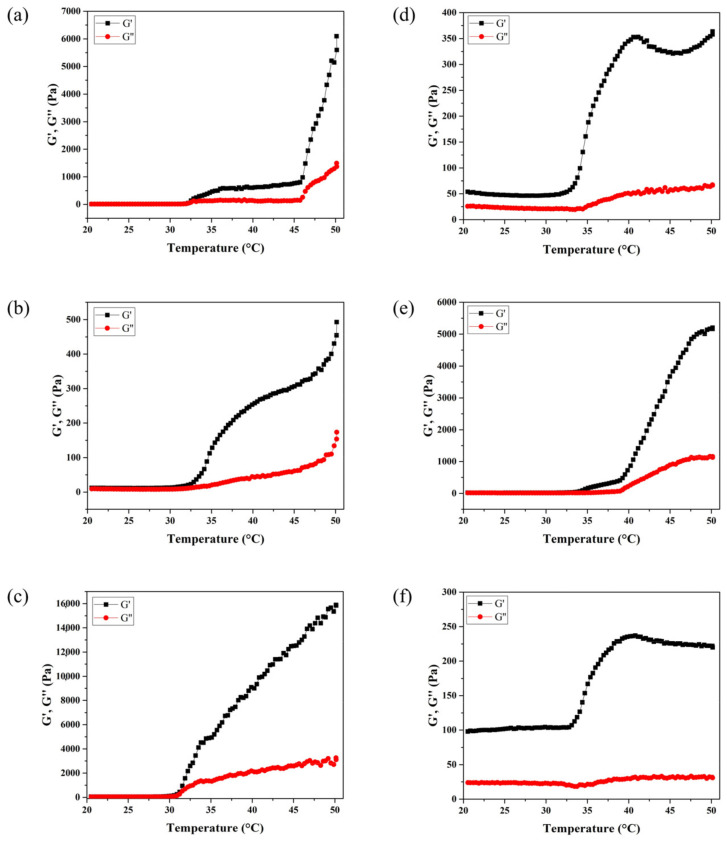
Oscillatory frequency sweeps of storage modulus, G′ (black curve), and the loss modulus, G″ (red curve) of thermo-responsive injectable hydrogel (**a**) PNIPAAm/Cs/CNC0, (**b**) PNIPAAm/Cs/CNC10, (**c**) PNIPAAm/Cs/CNC50, (**d**) PNIPAAm/Cs/CNC100, (**e**) PNIPAAm/Cs/CNC150 and (**f**) PNIPAAm/Cs/CNC300 at varied temperature 20–50 °C.

Figure 3 displays the rheological properties of hydrogel composites. As expected, they are responsive to temperature. While the loss modulus (G″) indicated the viscous (sol-like) behavior, the storage modulus (G′) depicted the elastic (gel-like) behavior of the material [35,37]. After the temperature was increased from 20 to 50 °C, all characteristic curves displayed the changes of G′ and G″. As the temperature increases to 30 °C, the storage modulus (G′) was found to increase dramatically, resulting in gel-like behavior. Furthermore, the sample PNIPAAm/Cs/CNC50 with optimum amount of CNCs showed highest G′ which is over 15,000 Pa, displaying the cooperative properties of CNCs in strengthening the hydrogel [43,44]. However, samples PNIPAAm/Cs/CNC100 and PNIPAAm/Cs/CNC300 (as shown in Figure 3d,f) have relatively lower values of G′ compared to the others, indicating lower mechanical strength of the hydrogels indicating that excessive amount of CNCs do not help with strengthening the hydrogel [37,45,46].

**Figure 4:**
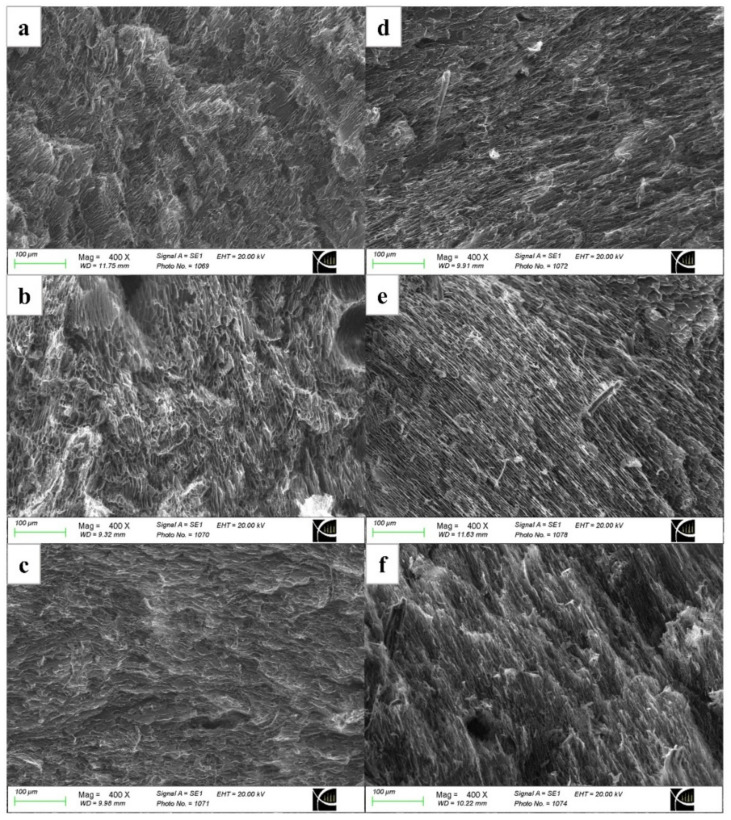
Scanning Electron Microscopy (SEM) images showing the microstructures of the composite hydrogels: (**a**) PNIPAAm/Cs/CNC0, (**b**) PNIPAAm/Cs/CNC10, (**c**) PNIPAAm/Cs/CNC50, (**d**) PNIPAAm/Cs/CNC100, (**e**) PNIPAAm/Cs/CNC150 and (**f**) PNIPAAm/Cs/CNC300. The cross-sectional view with a magnification of 400× is reported.

Figure 4 shows the microstructure of thermo-responsive composite hydrogels. The composite hydrogels (as shown in Figure 4b–f) revealed small pores in comparison to the sample without CNCs, Figure 4a. The increasing amount of CNC resulted in a reduction in pore sizes, which could be attributed to strong hydrogen bonding interactions throughout the network [47,48]. As a result of smaller pore sizes, the mechanical properties of the sample were significantly improved [49,50]. When an external load was applied, the presence of high quantity of smaller pores and micropores can be extremely beneficial to transfer load throughout the structure [51]. Furthermore, composite hydrogels with large amount of micropores and interconnected pores cell and tissue scaffolds. Hydrogels with microporous structure help with cell ingrowth, nutrient transport, waste removal, and a balanced mechanical strength that resembles native tissue [52].

**Figure 5:**
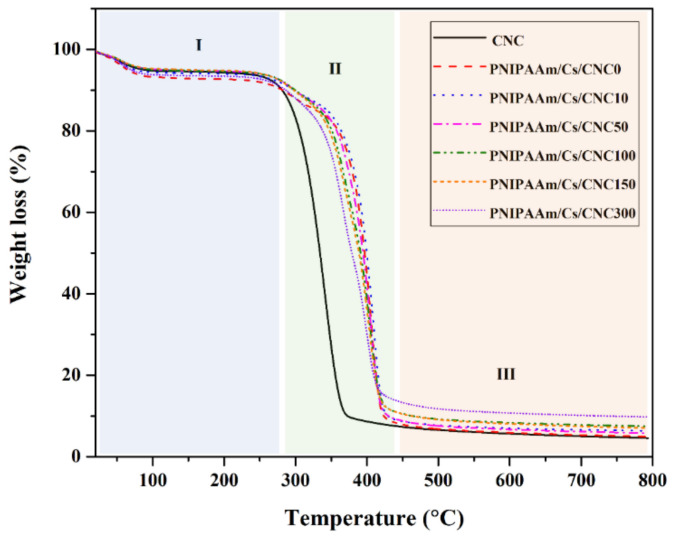
TGA thermogram of the composite hydrogel: PNIPAAm/Cs/CNC0, PNIPAAm/Cs/CNC10, PNIPAAm/Cs/CNC50, PNIPAAm/Cs/CNC100, PNIPAAm/Cs/CNC150 and PNIPAAm/Cs/CNC300. (I−III were the first stage, second stage and third stage of thermal decomposition, respectively).

Figure 5 shows the thermal properties of the composite hydrogels. The thermogram can be divided into three ranges. The first region of weight loss (%) from room temperature to 280 °C is attributed to the moisture elimination [53]. After that, the second range was between 280 to 430 °C. Composite hydrogels without CNC were found to decompose between 280–350 °C, while the samples with CNCs were found to degrade temperatures between 280 to 430 °C. This was probably due to high thermal stability of cellulose. The final stage of decomposition was shown above 430 °C. The curves in this region were characterized by plateau, which indicates minor or no weight change due to degradation. The remaining amounts (15% or less) were assigned to char and residues for all compositions.

**Figure 6:**
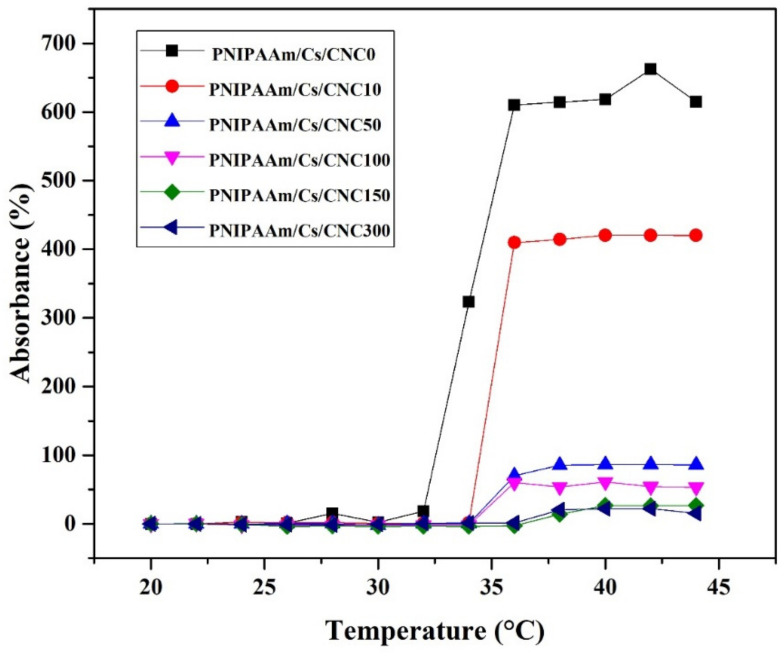
Thermo-responsive properties of the injectable composite hydrogel: PNIPAAm/Cs/CNC0, PNIPAAm/Cs/CNC10, PNIPAAm/Cs/CNC50, PNIPAAm/Cs/CNC100, PNIPAAm/Cs/CNC150 and PNIPAAm/Cs/CNC300.

The objective of volume phase transition temperature (VPTT) was to define the relationship between hydrogel stability and stimuli responsive behavior as a function of temperatures. Figure 6 reports the change in absorbance (%) of the thermo-responsive composite hydrogel at different temperatures. It is found that the sample without CNC was transparent at the initial stage. For the samples with CNCs and below the LCST, they were less transparent, and the transparency decreases with increasing amount of CNCs. When the temperature was increased, a shift in the absorbance (%) was observed reaching opacity at higher temperatures. The hydrogel solutions changed to opaque above the LCST temperature of 32 °C. Furthermore, the sol-gel transition has occurred around the body temperature as suggested by Khan et al. [38]. The VPTT of samples were typically noted as 34–38 °C. Interestingly, the VPTT of hydrogels is commonly lowered by adding hydrophobic comonomers and raised by incorporating hydrophilic comonomers [54]. The addition of CNC into the PNIPAAm-chitosan network shifted the hydrophobic/hydrophilic ratio in the hydrogel structure. The VPTT of the hydrogels were found to decrease by the addition of increasing amount of CNC.

**Figure 7:**
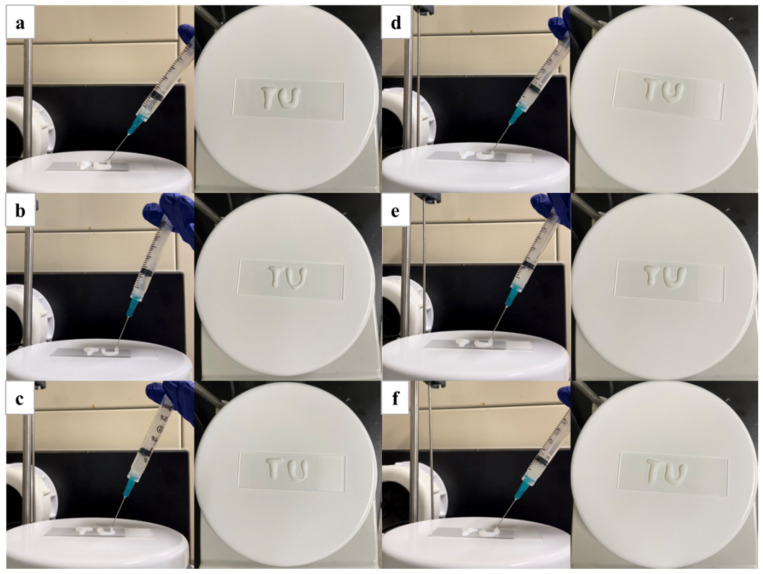
Injectability of the composite hydrogel (**a**) PNIPAAm/Cs/CNC0, (**b**) PNIPAAm/Cs/CNC10, (**c**) PNIPAAm/Cs/CNC50, (**d**) PNIPAAm/Cs/CNC100, (**e**) PNIPAAm/Cs/CNC150 and (**f**) PNIPAAm/Cs/CNC300.

The injectability of the composite hydrogels were also studied, as shown in Figure 7. All composite hydrogel solutions were placed into 23-gauge needles (23G) and then injected onto glass slides at 37 °C. The low viscosity of the hydrogel allowed easy delivery of the solution on the surface which subsequently hardened due to phase transition into a gel state at 37 °C [55,56,57]. At this temperature, the almost clear gel quickly changed to a white opaque gel.

### 3.2. Preliminary Investigation as a Medical Material

**Figure 8:**
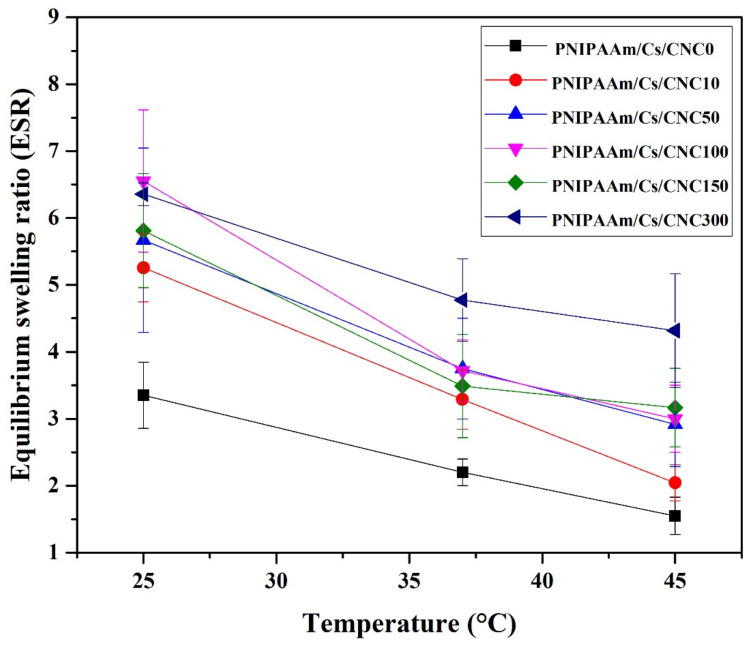
Equilibrium-swelling ratio of the injectable composite hydrogels: PNIPAAm/Cs/CNC0, PNIPAAm/Cs/CNC10, PNIPAAm/Cs/CNC50, PNIPAAm/Cs/CNC100, (PNIPAAm/Cs/CNC150 and PNIPAAm/Cs/CNC300 in PBS (pH 7.4) at 25, 37 and 45 °C.

Figure 8 shows the equilibrium-swelling ratio (ESR) of the composite hydrogels. When the temperature was increased, the ESR of the hydrogels was found to decrease. Below the LCST, the polymer chains are hydrated, allowing large amount of water molecules to be trapped within the hydrogel matrices of PNIPAAm, chitosan and CNCs [38,58]. However, above the LCST, the PNIPAAm chains become hydrophobic and dehydrated, leading to a collapse of polymeric chains [59]. It should be noted that high amount of CNCs significantly improved the equilibrium-swelling ratio of PNIPAAm/Cs/CNC composite hydrogel. The addition of CNCs increased the hydrophilicity of the hydrogel, and hence resulting in higher ESR [33,37,60].

**Figure 9:**
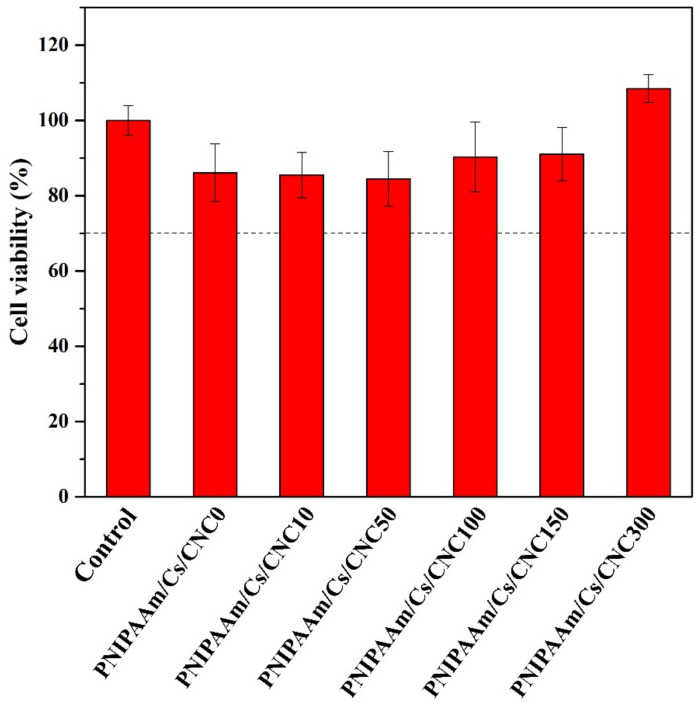
Cytotoxicity of the composite hydrogels: PNIPAAm/Cs/CNC0, PNIPAAm/Cs/CNC10, PNIPAAm/Cs/CNC50, PNIPAAm/Cs/CNC100, PNIPAAm/Cs/CNC150 and PNIPAAm/Cs/CNC300 with Human dermal fibroblast cells (HDFa).

The composite hydrogels were further evaluated for their in vitro toxicity using Human Dermal Fibroblast adult (HDFa) cells. As is shown in Figure 9, all PNIPAAm/Cs/CNC composite hydrogel showed 80% cell viability, which suggests that have low toxicity [61]. Furthermore, composite hydrogels with different amounts of CNCs showed lower toxicity as compared to composite hydrogel without CNCs. It has been reported that the varying concentrations of hemp-derived cellulose nanocrystals did not affect HDFa cells [33]. Due to their non-toxic and biocompatible characteristics, the composite hydrogels could be a promising biomaterial for wound healing and other applications [62,63].

## 4. Conclusions

The composite hydrogel based on Poly(*N*-Isopropylacrylamide), chitosan and cellulose nanocrystals was successfully fabricated by the photopolymerization. FT-IR spectra confirmed the incorporation of PNIPAAm, chitosan, and CNCs in the hydrogel structure. Rheological test demonstrated gelation within the temperature range of 20–50 °C, with a sharp increase in storage modulus (G′) above 30 °C, indicating gel-like behavior. The addition of CNCs reduced pore sizes to form smaller pores and micropores, contributing to enhanced mechanical properties. Thermal stability slightly decreased with increasing CNC content, but the volume phase transition temperature increased, ranging from 34–38 °C, close to physiological body temperature. Furthermore, all composite hydrogels can be injected with 23G needles at 37 °C, confirming their injectability. The equilibrium swelling ratio decreased with increasing temperature, but the presence of CNCs enhanced the swelling behavior. Furthermore, CNC incorporation improved cell viability, suggesting that PNIPAAm/Cs/CNC composite hydrogels are promising candidates for wound dressing and healing applications.

## Data Availability

Data is contained within the article.
